# Na^+^,K^+^-ATPase with Disrupted Na^+^ Binding Sites I and III Binds Na^+^ with Increased Affinity at Site II and Undergoes Na^+^-Activated Phosphorylation with ATP

**DOI:** 10.3390/biom14010135

**Published:** 2024-01-22

**Authors:** Hang N. Nielsen, Rikke Holm, Ryan Sweazey, Jens Peter Andersen, Pablo Artigas, Bente Vilsen

**Affiliations:** 1 Department of Biomedicine, Aarhus University, DK-8000 Aarhus, Denmark; 2 Department of Cell Physiology and Molecular Biophysics, Center for Membrane Protein Research, Texas Tech University Health Sciences Center, Lubbock, TX 79430, USApablo.artigas@ttuhsc.edu (P.A.)

**Keywords:** Na^+^,K^+^-pump, Na^+^ site, K^+^ site, Na^+^ affinity, K^+^ affinity, P-type ATPase, mutagenesis, serine, phosphorylation, electrophysiology

## Abstract

Na^+^,K^+^-ATPase actively extrudes three cytoplasmic Na^+^ ions in exchange for two extracellular K^+^ ions for each ATP hydrolyzed. The atomic structure with bound Na^+^ identifies three Na^+^ sites, named I, II, and III. It has been proposed that site III is the first to be occupied and site II last, when Na^+^ binds from the cytoplasmic side. It is usually assumed that the occupation of all three Na^+^ sites is obligatory for the activation of phosphoryl transfer from ATP. To obtain more insight into the individual roles of the ion-binding sites, we have analyzed a series of seven mutants with substitution of the critical ion-binding residue Ser777, which is a shared ligand between Na^+^ sites I and III. Surprisingly, mutants with large and bulky substituents expected to prevent or profoundly disturb Na^+^ access to sites I and III retain the ability to form a phosphoenzyme from ATP, even with increased apparent Na^+^ affinity. This indicates that Na^+^ binding solely at site II is sufficient to promote phosphorylation. These mutations appear to lock the membrane sector into an E_1_-like configuration, allowing Na^+^ but not K^+^ to bind at site II, while the cytoplasmic sector undergoes conformational changes uncoupled from the membrane sector.

## 1. Introduction

**Scheme 1:**
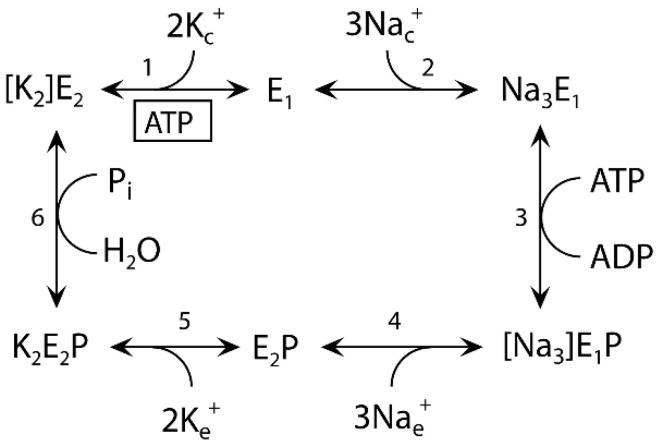
Na^+^,K^+^-ATPase reaction cycle. E_1_ and E_2_ represent the main conformational states of the enzyme. P indicates phosphorylation. E_1_P and E_2_P are the respective ADP-sensitive and ADP-insensitive (K^+^ sensitive) phosphoenzyme intermediates. Occluded Na^+^ and K^+^ ions are shown in brackets. Free ions are labeled c and e for the cytoplasmic and extracellular side, respectively. Boxed ATP indicates ATP bound in a non-phosphorylating mode, enhancing the rate of K^+^ deocclusion and accompanying E_2_–E_1_ conformational change. The partial reactions of the cycle are numbered 1–6 (the forward running mode is clockwise).

Na^+^,K^+^-ATPase, also known as the Na^+^,K^+^-pump, is a membrane-associated ion-transporting enzyme belonging to the P-type ATPase family. It is responsible for creating gradients for Na^+^ and K^+^ across the plasma membranes of animal cells, fundamental to a variety of physiological processes [1,2,3,4]. Insight into the structure–function relationship and molecular mechanism of the Na^+^,K^+^-pump has been advanced through the determination of the atomic structures of the protein using X-ray crystallography and cryo-electron microscopy [5,6,7,8,9]. Na^+^,K^+^-ATPase consists of α-, β-, and γ-subunits, with the α-subunit containing the ATP- and ion-binding sites located in the cytoplasmic and transmembrane domains, respectively. During its functional cycle, Na^+^,K^+^-ATPase actively extrudes three cytoplasmic Na^+^ ions in exchange for two K^+^ ions from the extracellular side for each ATP hydrolyzed, as described using the Post–Albers model illustrated in Scheme 1, in which a sequential binding and release of the ions occurs, with Na^+^ being translocated before K^+^ in each cycle [2,10]. The transport process is defined by four major conformational states: the dephosphoenzyme forms, E_1_ and E_2_, and the phosphoenzyme forms, E_1_P and E_2_P. The transitions between these conformational states are combined with alterations in selectivity for the ions and a change in orientation of the ion-binding sites with respect to the membrane. E_1_ is Na^+^-selective and has the ion-binding pocket facing the cytoplasm. E_1_P has the three Na^+^ ions bound in an occluded state. E_2_P is K^+^-selective, although it can bind Na^+^ with low affinity, and has the binding pocket facing the external side. E_2_ has two K^+^ ions bound in an occluded state, cf. Scheme 1 [2,3,4,5,6,7,11].

The membrane domain of the α-subunit consists of 10 transmembrane helices (M1-M10), of which M4, M5, M6, and M8 contribute ion-coordinating residues to the binding of Na^+^ and K^+^ [5,6,7,12,13,14,15,16,17]. Using the nomenclature originating from the first structure determination of the closely related Ca^2+^-ATPase [18,19], the three Na^+^ sites are denoted as I, II, and III (with I and II corresponding to Ca^2+^ sites I and II in Ca^2+^-ATPase). The Na^+^ sites I and II overlap with the two K^+^ sites, comprising almost the same residues in the two different conformations, E_1_ and E_2_, respectively. Site III is only present in the E_1_ form and is unique and Na^+^-specific. When the three sites of E_1_ are occupied by Na^+^, the enzyme undergoes phosphorylation from ATP (reaction 3 in Scheme 1). Although the E_1_ form binds K^+^ at sites I and II, the K^+^-bound E_1_ form is unable to undergo phosphorylation and is unstable, being transformed into the K^+^-occluded [K_2_]E_2_ form (reaction 1 in Scheme 1, backward direction of the cycle).

The sequence of filling the three sites, when Na^+^ binds from the cytoplasmic side, has been controversial. Studies with electrochromic dyes have led to the view that the last Na^+^ to bind is the one at site III [11]; however, based on the crystal structure, it has been proposed that site III is the first to be occupied and site II the last [7].

**Figure 1:**
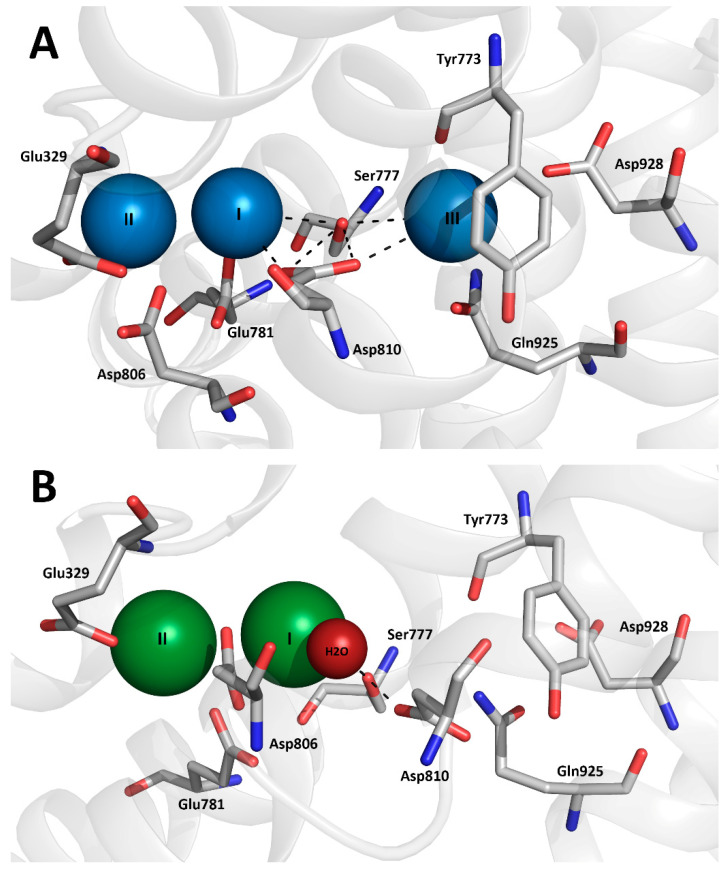
Na^+^ and K^+^ binding sites in Na^+^,K^+^-ATPase crystal structures with indication of Ser777 and other residues relevant to ion binding. (**A**): The Na^+^-bound [Na_3_]E_1_·AlF_4_^−^·ADP form (mimicking the transition state between Na_3_E_1_ and [Na_3_]E_1_P, cf. Scheme 1), Protein Data Bank code 3WGV, chain A (so-called protomer B) [7]. Na^+^ ions are shown as blue spheres numbered I, II, and III. (**B**): The K^+^-bound [K_2_]E_2_·MgF_4_^2–^ form (mimicking [K_2_]E_2_·P_i_ between K_2_E_2_P and [K_2_]E_2_ in Scheme 1), Protein Data Bank code 2ZXE [6]. K^+^ ions are shown as green spheres numbered I and II, and the associated water molecule in red. Both structures are viewed along the plane of the membrane with the cytoplasmic side up. Selected residues contributing to ion coordination are shown in stick representation colored according to the elements (carbon, gray; oxygen, red; nitrogen, blue) and numbered according to the rat α1-isoform. Broken lines indicate potential hydrogen bonds or bonds between ions and oxygen ligands of the side chains of Ser777 and Asp810.

In the present study, we focus on the M5 serine residue Ser777 (rat α_1_ numbering of residues is used throughout this paper; in the pig α_1_ enzyme for which the atomic structure with bound Na^+^ has been obtained [7], the numbers differ from rat α_1_ by −2). In the model for Na^+^ binding to E_1_ proposed based on the crystal structure, the side chain of this serine residue plays a crucial role in receiving the first and the second Na^+^ ions, binding at sites III and I, respectively [7]. The side chain oxygen atom of Ser777 functions as a shared ligand of the two Na^+^ ions at these sites and is positioned for the optimal binding of the second Na^+^ as a consequence of its interaction with the first Na^+^, thereby contributing to the cooperativity of the Na^+^ binding (cf. Figure 1A). In previous mutagenesis studies of this serine in Na^+^,K^+^-ATPase, replacing it with alanine, wild type-like or only slightly modified Na^+^ binding properties of the S777A mutant were reported in two studies [13,14], while a third study [16] indicated a larger effect of the S777A mutation on the Na^+^ binding. The importance of this serine in Na^+^ binding at the cytoplasmic-facing sites in E_1_ conformation is further underscored by the finding that the replacement of the lysine at the corresponding position in non-gastric H^+^,K^+^-ATPase with alanine or serine confers the ability to bind Na^+^, albeit with very low affinity, at sites I and III [20]. In relation to this residue, it is moreover of interest that the mutation of the α3-isoform of Na^+^,K^+^-ATPase corresponding to S777R has been identified as pathogenic in a patient with the neurological disorder alternating hemiplegia of childhood [17,21], and the mutation corresponding to S777N in the α2-isoform has been associated with hypokalemic periodic paralysis [22].

From the K^+^-bound E_2_ crystal structure, Ser777 likewise appears to be a crucial residue, involved directly in binding the K^+^ at site I, as well as indirectly through the binding (together with Asp810) of a water molecule interacting with the K^+^ ion (Figure 1B) [6].

The purpose of the present study was, using functional analysis of a series of seven Ser777 mutants, to obtain more insight into the individual roles of the ion-binding sites in the function of Na^+^,K^+^-ATPase. We have studied mutants with Ser777 being replaced by residues of varying size, anticipating that because the serine is part of both sites I and III, a large and bulky side chain at its position might disrupt these sites and leave site II relatively intact, thus allowing the function of site II to be addressed separately. We found that only the Ser777 mutants with the smallest substituents were transport-active. However, we were able to study the partial reactions of all seven mutants and obtain information on the ability of Na^+^ and K^+^ to activate phosphorylation and dephosphorylation, respectively. Surprisingly, the largest side chains allowed phosphorylation with an increased apparent affinity for Na^+^, indicating that the occupation of site II alone with Na^+^ is sufficient to trigger phosphorylation.

## 2. Materials and Methods

### 2.1. Site-Directed Mutagenesis and Expression

Point mutations corresponding to the amino acid substitutions S777G/T/N/Q/V/L/Y were introduced into full-length cDNA encoding the ouabain-resistant rat α1-isoform of Na^+^,K^+^-ATPase using PCR [23]. The resulting constructs were full-length-sequenced to verify the correct point mutation, both before transfection into COS-1 cells using the Ca^2+^-phosphate precipitation method [24] and after expanding single colonies into stable cell lines under ouabain selection pressure [12,23,25]. The generation of stable cell lines using the ouabain selection method was only feasible for the mutants S777G, S777T, and S777N, as well as the wild type. The remaining mutants, S777Q/V/L/Y, were unable to support cell growth. Because they were expressed in the plasma membrane, as evidenced in the phosphorylation experiments described below, the inability to support cell growth must be a consequence of a lack of sufficient transport activity rather than a lack of expression. Generally, rat α1-isoform mutants with less than 5% transport activity are unable to support the growth of COS-1 cells under ouabain selection pressure [17]. Hence, the mutants S777Q/V/L/Y are, in the following, denoted as “inactive mutants”. These mutants could be expressed transiently by the previously described strategy [23], combining the Ca^2+^-phosphate precipitation method with siRNA to silence the endogenous Na^+^,K^+^-ATPase. Thereby, we were able to study the partial reactions of these mutant enzymes despite their inability to perform a full Na^+^,K^+^ transport cycle. For comparison, the wild type was also expressed transiently using the siRNA strategy.

### 2.2. Isolation of the Plasma Membrane Vesicles and ATPase Activity Measurements

Prior to performing the functional analysis, the plasma membrane fractions containing the recombinant wild-type enzyme or recombinant mutants were isolated from the COS-1 cells using differential centrifugation and made leaky with either sodium deoxycholate (MERCK, Darmstadt, Germany) or alamethicin (Sigma, Darmstadt, Germany), 0.65 mg/mL and 0.14 μg/mL, respectively, at a protein concentration of 0.4 mg/mL, to give access to the ATP and ions from both sides of the membrane [23]. The ATPase activity of the active mutants, as well as the wild type, expressed in the stable cell lines was determined in the leaky membrane preparation using the colorimetric procedure of Baginski [23,26]. The liberation of P_i_ was followed at 37 °C for 15–19 min (i.e., within a linear time range) in the presence of 30 mM histidine (Sigma-Aldrich, Darmstadt, Germany), 1 mM EGTA (Sigma-Aldrich, Steinheim, Germany), 3 mM MgCl_2_ (MERCK, Darmstadt, Germany), 3 mM ATP (MERCK, Darmstadt, Germany), 10 μM ouabain (Sigma, Darmstadt, Germany) inhibiting the endogenous COS-1 cell Na^+^,K^+^-ATPase, and various concentrations of NaCl (VWR, Leuven, Belgium) and KCl (MERCK, Darmstadt, Germany), allowing the determination of the concentration dependencies of these ligands. The Na^+^ dependence of the ATPase activity in the absence of K^+^ (“Na^+^-ATPase activity”) was determined in the same way without K^+^ present. The protein concentration during the assay was 20 μg/mL. For background subtraction, similar measurements were carried out in the presence of 10 mM ouabain to inhibit all Na^+^,K^+^-ATPase-related ATPase activity. The specific ATPase activity was expressed in nmol ATP hydrolyzed/min/mg plasma membrane protein and was normalized to the maximum value (taken as 100%) to visualize the difference in Na^+^ affinity, rather than the maximal specific activity. For comparison of the maximal activities per expressed Na^+^,K^+^-ATPase protein, the catalytic turnover rate (in min^−1^) was calculated as the ratio between the specific ATPase activity (nmol ATP hydrolyzed/min/mg plasma membrane protein) and the active site concentration (nmol/mg plasma membrane protein) determined using phosphorylation under stoichiometric conditions, as described in Section 2.3 below (a measure of the expression level per mg plasma membrane protein).

### 2.3. Phosphorylation Experiments

Phosphorylation studies were performed on the leaky membrane preparation according to the previously established principles [23]. To determine the Na^+^ dependence of the phosphorylation, the membranes were incubated for 10 s at 0 °C with 2 µM [γ-^32^P]ATP (3000 Ci/mmol, Revvity, Boston, MA, USA, obtained via Lier, Belgium, mixed with non-radioactive ATP (MERCK, Darmstadt, Germany) to give a final activity of approximately 0.12 MBq/mL) in a medium containing 20 mM Tris (Sigma, Steinheim, Germany), pH 7.5, 3 mM MgCl_2_ (MERCK, Darmstadt, Germany), 1 mM EGTA (Sigma-Aldrich, Darmstadt, Germany), 20 µg oligomycin/mL (Sigma-Aldrich, Schelldorf, Germany) (to prevent dephosphorylation), 10 or 100 µM ouabain (Sigma, Darmstadt, Germany), and various concentrations of NaCl (VWR, Leuven, Belgium) together with a varying concentration of *N*-methyl-D-glucamine (NMDG+) (FLUKA, Steinheim, Germany) maintaining the ionic strength. For examination of the stably expressed mutants, the medium contained 10 µM ouabain, whereas the ouabain concentration was increased to 100 µM for the transiently expressed mutants to make sure that the endogenous enzyme (making up a larger fraction of the total amount of Na^+^,K^+^-ATPase in this case) was knocked completely out. The fact that the apparent affinities for Na^+^ and K^+^ obtained with the stably and transiently expressed wild type are identical (see Table 1) documents that the difference in ouabain concentration does not influence the results. The phosphorylation was quenched with ice-cold 1 M phosphoric acid (MERCK, Darmstadt, Germany), pH 2.4. In these and the below-described phosphorylation experiments, the acid-precipitated ^32^P-labeled phosphoenzyme was washed using centrifugation prior to SDS (MERCK, Darmstadt, Germany) polyacrylamide gel electrophoresis at a pH 6.0 of for 15 min at room temperature, followed by approximately 1 h at 15 °C to prevent any degradation of the enzyme due to heat development. The visualization and quantification of the separated ^32^P-labeled phosphoenzyme band were obtained using phosphor imaging using a Cyclone Plus Storage System (Model C431200, PerkinElmer, Waltham, MA, USA).

To determine the apparent affinity for K^+^ inhibition of phosphorylation, the leaky membranes were incubated with 2 µM [γ-^32^P]ATP (3000 Ci/mmol, Revvity, Boston, MA, USA, obtained via Lier, Belgium, mixed with non-radioactive ATP (MERCK, Darmstadt, Germany) to give a final activity of approximately 0.12 MBq/mL) for 10 s at 0 °C in a medium containing 20 mM Tris (Sigma, Steinheim, Germany), pH 7.5, 50 mM NaCl, 3 mM MgCl_2_ (MERCK, Darmstadt, Germany), 1 mM EGTA (Sigma-Aldrich, Darmstadt, Germany), 10 or 100 µM ouabain (as explained for Na^+^ dependence above), and various concentrations of KCl (MERCK, Darmstadt, Germany), together with a varying concentration of choline chloride (Sigma, Steinheim, Germany) to maintain the ionic strength. Acid quenching was carried out as described above for the Na^+^ dependence of phosphorylation.

The E_1_P–E_2_P distribution of the phosphoenzyme was determined by adding ADP to the phosphorylated enzyme to dephosphorylate the ADP-sensitive fraction (E_1_P). Phosphorylation was carried out using incubation of the leaky membranes with 2 µM [γ-^32^P]ATP (activity approximately 0.12 MBq/mL) for 5 s at 0 °C in a medium containing 20 mM Tris (pH 7.5), 50 mM NaCl, 3 mM MgCl_2_, 1 mM EGTA, and 10 or 100 µM ouabain (as explained for Na^+^ dependence above). The chemicals used in this assay were the same as described previously in this section. To induce dephosphorylation, 2.5 mM ADP (Sigma, Steinheim, Germany) with 1 mM ATP (non-radioactive) was added, followed by acid quenching at various times.

The active site concentration was determined using phosphorylation with [γ-^32^P]ATP under stoichiometric conditions, i.e., as described above for the Na^+^ dependence of phosphorylation in the presence of 150 mM NaCl with 20 µg oligomycin/mL to prevent dephosphorylation [23].

### 2.4. Electrophysiological Experiments

Oocytes were extracted from *Xenopus* toads in accordance with the approved TTUHSC IACUC protocols and enzymatically dissociated [23]. Upon dissociation, the oocytes were kept in SOS solution composed of (in mM) 100 NaCl, 1 MgCl_2_, 2 KCl, 1.8 CaCl_2_, 5 HEPES, 2.5 pyruvic acid (all from Sigma, Saint Louis, MO, USA), 1x antibiotic-antimycotic (Gibco, Grand Island, NY, USA), and 5% horse serum (Gibco, Auckland, New Zealand), titrated to a pH of 7.5 with NaOH (Sigma, Saint Louis, MO, USA). The mutation S777V was introduced into a Xenopus α1 template that had been made ouabain-insensitive by introducing Q120R and N131D (equivalent to rat α1 R113 and D124, respectively), which confer ouabain resistance to the pump by mimicking the residues responsible for ouabain insensitivity in the rat α1-isoform. For simplicity, throughout the manuscript, we use rat α1 numbering (7 lower than Xenopus α1). Both α1 and β3 (in the pSD5 vector) were linearized using the Bgl*II* restriction enzyme (Thermo Fischer, Vilnius, Lithuania) and in vitro-transcribed using the SP6 mMESSAGE mMACHINE kit (Invitrogen, Vilnius, Lithuania). The oocytes were injected with cRNA mixtures of α1:β1 (75 ng α1, 25 ng β1) and kept at 16 °C until recording (3–6 days). Two-electrode voltage clamp was performed using glass microelectrodes (0.2–1 MΩ, filled with 3 M KCl) and an OC-725C amplifier (Warner Instruments, Holliston, MA, USA), controlled using the pCLAMP software (pCLAMP Software Suite version 11) and using a Digidata 1440 (both from Molecular Devices, San Jose, CA, USA). The data were acquired at 10 kHz and were continuously recorded using a MiniDigi 1A (Molecular Devices, San Jose, CA, USA) at 1 kHz. Before recording, the oocytes were incubated for 1 h in a Na^+^-loading solution containing (in mM, all chemicals from Sigma, Saint Louis, MO, USA): 90 NaOH, 20 tetraethylammonium (TEA)-OH, 40 HEPES, and 0.2 EGTA, titrated to a pH of 7.2 with sulfamic acid (~220 mOsm/kg), with 10 μM ouabain to inhibit endogenous pumps. After incubation, the oocytes were kept in a 125 mM Na^+^ recording solution until recording.

The recording solutions contained (in mM, all chemicals from Sigma, Saint Louis, MO, USA): 133 methanesulfonic acid (MS), 5 Ba(OH)_2_, 1 Mg(OH)_2_, 0.5 Ca(OH)_2_, 10 HEPES, and 125 of either NaOH, NMDG^+^, or KOH, titrated to a pH of 7.6 with MS (~260 mOsm/kg). Ouabain was dissolved directly in the external solutions at 10 mM.

### 2.5. Data Analysis and Statistics

The data points shown in the figures are mean ± SEM values (indicated by error bars). The data in the table are mean values ± SD values, with the number of independent experiments indicated. All experiments were performed independently, and different preparations of isolated plasma membranes were used for each mutant and wild type. The SigmaPlot software (version 10.0, SYSTAT, Palo Alto, CA, USA) was used to fit the relevant equations to the data points using non-linear regression. The Na^+^ dependence of the phosphorylation and ATPase activity (normalized to the maximal value to obtain %) was fitted using a Hill function (Equation (1)):

(1) $$A=A_{max}\cdot \left(\frac{{\left[L\right]}^{n}}{K_{0.5}^{n}+{\left[L\right]}^{n}}\right)$$

*A* represents the actual phosphorylation level or ATPase activity (in %) at the given ligand (L) concentration, here Na^+^; *A_max_* is the extrapolated maximum value corresponding to infinite ligand concentration; *K*_0.5_ is the ligand concentration giving half-maximum activation (reciprocal of apparent affinity); and *n* is the Hill coefficient.

In cases where the Na^+^ dependence of the ATPase activity showed inhibition at a high substrate concentration, the inhibition was represented using a negative Hill function (Equation (2)):

(2) $$A=\frac{A_{max}{\left[L\right]}^{n1}}{\left(K_{0.5A}^{n1}+{\left[L\right]}^{n1}\right)}-\frac{B_{max}{\left[L\right]}^{n2}}{\left(K_{0.5B}^{n2}+{\left[L\right]}^{n2}\right)}$$

The activation and inhibition phases are indexed as *A* and *B*, respectively, while *n*1 and *n*2 are the corresponding Hill coefficients.

The K^+^ inhibition of phosphorylation was fitted using a Hill function modified to represent ligand-induced inhibition (Equation (3)):

(3) $$A=A_{0}+A_{max}\cdot \left(1-\frac{{\left[L\right]}^{n}}{K_{0.5}^{n}+{\left[L\right]}^{n}}\right)$$

*A* represents the actual phosphorylation level at the given ligand (L) concentration, here K^+^, and *A*_0_ is the phosphorylation level corresponding to an infinite K^+^ concentration, i.e., the maximal inhibition. *A_max_* is the maximal value of the variable fraction corresponding to a zero concentration of K^+^. *K*_0.5_ is the K^+^ concentration that gives half-maximum inhibition (reciprocal of apparent affinity) and *n* is the Hill coefficient.

The distribution of the phosphoenzyme between E_1_P (ADP-sensitive) and E_2_P (ADP insensitive) was determined by fitting a double exponential decay function to the dephosphorylation time course obtained upon the addition of ADP (Equation (4)):

(4) $$EP=E_{1}P\cdot {exp}^{\left(-k_{1}t\right)}+E_{2}P\cdot {exp}^{\left(-k_{2}t\right)}$$

EP is the total amount of phosphoenzyme. E_1_P and E_2_P are the two phosphoenzyme intermediates that are ADP-sensitive and ADP-insensitive, respectively. *k*_1_ and *k*_2_ are the decay constants for the E_1_P and E_2_P phases, respectively.

Transient currents elicited by 200 ms long pulses were integrated (after baseline correction to eliminate constant current), both during the pulse and after the pulse was turned off. The Q–V charge–voltage curves were fitted with a Boltzmann distribution (Equation (5)):

Q = Q_hyp_ + Q_tot_/(1 + *exp*[z_q_e(V − V_0.5_)/kT]) (5)

Q_hyp_ is the charge moved by the hyperpolarizing pulses, Q_tot_ is the total charge moved, V_0.5_ is the center of the distribution, z_q_ is the valence of a charged particle if it crossed the entire membrane electric field, e is the elementary charge, k the Boltzmann constant, and T the temperature. kT/z_q_e is sometimes also referred to as the slope factor, as it relates to the steepness of the curve.

## 3. Results

### 3.1. Expression of the Ser777 Mutants

First, we attempted to obtain stable transfectants of all the Ser777 mutants S777G/T/N/Q/V/L/Y, as well as the wild type, taking advantage of the ouabain selection methodology, which is feasible using the ouabain-insensitive rat α_1_ enzyme [23,25]. Stable transfectants could be obtained in the presence of 1–5 μM ouabain for the wild type and the mutants S777G, S777T, and S777N. For the mutants, it was necessary to supplement the DMEM growth medium with KCl to obtain a final concentration of 20 mM KCl (DMEM has 5.4 mM KCl), which already at this stage indicated a reduced affinity for extracellular K^+^. Under the same conditions, the mutants S777Q, S777V, S777L, and S777Y did not support cell growth, indicating that they are inactive in Na^+^ and/or K^+^ transport. For these S777 mutants, we used a combination of transient transfection and siRNA methodology to knock down the endogenous Na^+^,K^+^-ATPase of the COS-1 cells [23], thereby reducing the background to a level that allowed the examination of the partial reaction steps of these mutants, although they were inactive with respect to the overall ATPase reaction.

### 3.2. Na^+^,K^+^-ATPase Activity and Na^+^ Dependence of the Active Mutants S777G, S777T, and S777N

**Figure 2:**
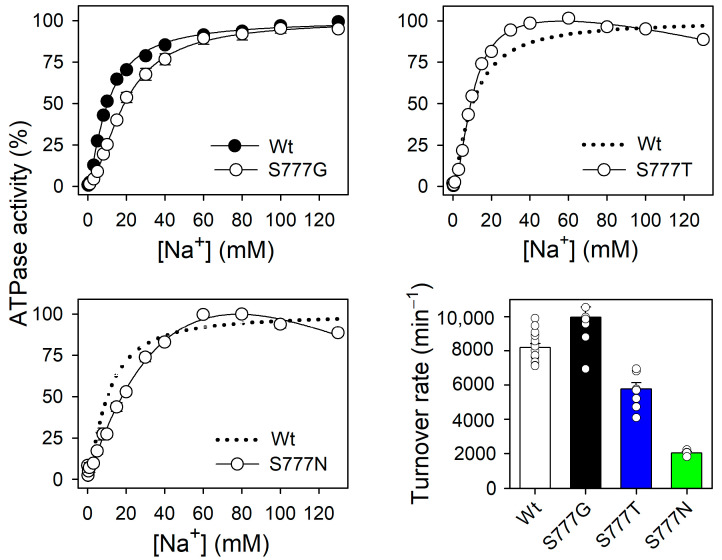
Na^+^ dependence of Na^+^,K^+^-ATPase activity and catalytic turnover rate. ATPase activity was measured in the presence of 20 mM K^+^ at the indicated concentrations of Na^+^ (130 mM for the turnover rate in the column diagram). Symbols with error bars (seen only when larger than the size of the symbols) represent mean ± SEM. For the Na^+^ dependence, the data were normalized corresponding to a maximum of 100% in each case. Each line represents the best fit of the Hill function with or without a second term representing inhibition (Equations (1) and (2) of Section 2.5). Extracted *K*_0.5_ values are listed in Table 1 with statistics. Dotted lines reproduce the wild type for direct comparison in the same panel. The catalytic turnover rate (column diagram) was calculated by relating the Na^+^,K^+^-ATPase activity determined in the presence of 130 mM Na^+^ and 20 mM K^+^ to the active site concentration determined using phosphorylation from [γ-^32^P]ATP under stoichiometric conditions (see Section 2.3).

For the wild type and the three active mutants, S777G, S777T, and S777N, showing measurable Na^+^,K^+^-ATPase activity, the turnover rate was determined at 130 mM Na^+^ and 20 mM K^+^ by relating the specific Na^+^,K^+^-ATPase activity measured under these conditions to the active site concentration determined using phosphorylation from [γ-^32^P]ATP under stoichiometric conditions. In this way, any variation in the specific activity caused by a difference in the expression levels is eliminated. The turnover rate was wild-type-like for S777G, whereas it was moderately (1.5-fold) and markedly (4-fold) reduced for the S777T and S777N mutants, respectively (Figure 2, column diagram, and Table 1).

The apparent affinity for Na^+^ activation of the Na^+^,K^+^-ATPase activity at a constant K^+^ concentration of 20 mM (Figure 2 and Table 1) was reduced (*K*_0.5_ increased) for S777G and S777N (~2-fold), relative to the wild type, and was wild-type-like for the S777T mutant. The apparent affinity determined under these conditions depends on the binding of Na^+^ to the sites that, in intact cells, face the cytoplasm, as well as on the competition between the Na^+^ and K^+^ at these sites and the cycling of the enzyme through multiple conformational states. For S777T and S777N, the ATPase activity at the highest Na^+^ concentration of 130 mM was slightly lower than the maximum activity at 60–80 mM Na^+^. For the wild type, such Na^+^-dependent inhibition at a high Na^+^ concentration is only seen at K^+^ concentrations significantly lower than the 20 mM K^+^ present here. This inhibition reflects the competitive binding of Na^+^ at the extracellular-facing sites where K^+^ normally promotes dephosphorylation [23]. Hence, the inhibition seen for S777T and S777N indicates that the ratio between the Na^+^ and K^+^ affinities at the extracellular-facing sites is shifted in favor of Na^+^, i.e., their K^+^ selectivity is reduced.

**Table 1:** Results of the biochemical analysis of wild type and mutants. Mean values ± SD are shown, with the number of independent experiments in parentheses. For *K*_0.5_ values, the relevant equation (Section 2.5) was fitted to each independent data set, and the mean value was calculated. ND, not determined.

	Turnover Rate	Na^+^ Activation		K^+^ Inhibition	E_2_P
		ATPase Activity *K*_0.5_ (Na^+^)	Phosphorylation *K*_0.5_ (Na^+^)	Phosphorylation *K*_0.5_ (K*^+^*)	
	min^−1^	mM			(%)
Wt (stable)	8187 ± 227	10 ± 0.8	0.50 ± 0.10	0.052 ± 0.026	62 ± 5
	(n = 15)	(n = 6)	(n = 13)	(n = 6)	(n = 7)
Wt (transient)	ND	ND	0.47 ± 0.09	0.050 ± 0.028	61 ± 7
			(n = 20)	(n = 21)	(n = 15)
S777G	9965 ± 1698	19 ± 2	3.03 ± 0.57	0.563 ± 0.105	90 ± 8
	(n = 8)	(n = 3)	(n = 4)	(n = 4)	(n = 4)
S777T	5760 ± 1057	9.3 ± 0.2	0.72 ± 0.13	1.14 ± 0.16	75 ± 3
	(n = 8)	(n = 3)	(n = 4)	(n = 3)	(n = 3)
S777N	2041 ± 166	18 ± 2	3.23 ± 0.24	1.96 ± 0.67	87 ± 3
	(n = 4)	(n = 3)	(n = 3)	(n = 3)	(n = 3)
S777Q	ND	ND	0.17 ± 0.05	>50	86 ± 3
			(n = 5)		(n = 3)
S777V	ND	ND	0.049 ± 0.044	>50	100 ± 8
			(n = 6)		(n = 4)
S777L	ND	ND	0.068 ± 0.019	>50	83 ± 7
			(n = 5)		(n = 5)
S777Y	ND	ND	0.018 ± 0.019	>50	72 ± 3
			(n = 3)		(n = 3)

### 3.3. Na^+^ Dependence of Phosphorylation in All Ser777 Mutants

**Figure 3:**
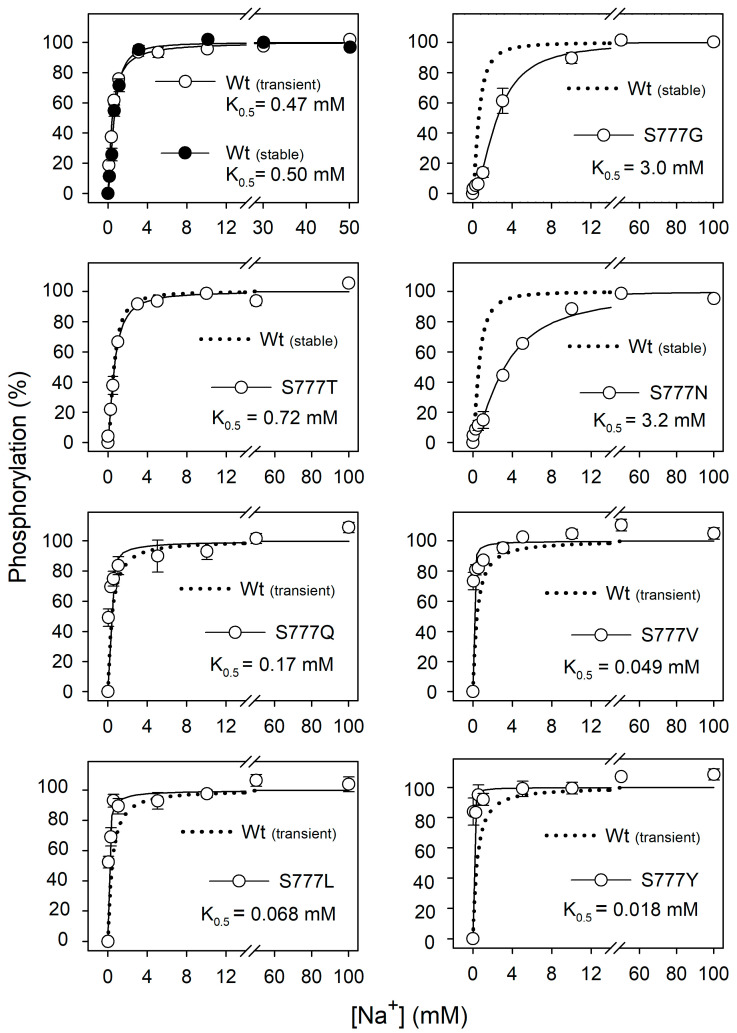
Na^+^ dependence of phosphorylation. The enzyme was phosphorylated with [γ-^32^P]ATP at the indicated concentrations of Na^+^ in the presence of oligomycin to inhibit dephosphorylation. For further details, see Section 2.3. Symbols with error bars (seen only when larger than the size of the symbols) represent mean ± SEM. Each line represents the best fit of a Hill function (Equation (1) of Section 2.5). Extracted *K*_0.5_ values are shown in the panels and are listed in Table 1 with statistics. Data for both the stably expressed wild type and the transiently expressed wild type are shown. Dotted lines reproduce in each panel the wild type expressed in the same way as the mutant for direct comparison.

To address the Na^+^ binding at the cytoplasmic-facing sites in the absence of interference from K^+^ and the cycling of the enzyme, we studied the Na^+^ dependence of the phosphorylation with [γ-^32^P]ATP (Scheme 1, reactions 2 and 3) in the presence of oligomycin, which inhibits the E_1_P-to-E_2_P transition, thus stabilizing the phosphoenzyme. Figure 3 and Table 1 show that all the S777 mutants, including the inactive mutants S777Q/V/L/Y, were able to become phosphorylated, thus allowing an assessment of their Na^+^ affinity. For S777G and S777N, the apparent Na^+^ affinity was markedly reduced (6- to 7-fold), relative to the wild type, whereas S777T displayed a minor (1.4-fold) reduction. These data are in good agreement with the results described above for the Na^+^,K^+^-ATPase activity, but reveal a larger effect on Na^+^ binding when K^+^ is absent and the cycling of the enzyme is prevented. Surprisingly, the inactive mutants S777Q/V/L/Y all displayed a markedly enhanced apparent affinity for Na^+^ (*K*_0.5_ reduced). The polar glutamine substituent, which deviates only from asparagine by the side chain being one carbon atom longer, showed a three-fold increase in apparent Na^+^ affinity. The larger and hydrophobic substituents valine, leucine, and tyrosine increased the apparent Na^+^ affinity 7- to 26-fold, which was most pronounced for the tyrosine, despite the presence of a hydroxyl group, as in serine.

### 3.4. K^+^ Inhibition of Phosphorylation

**Figure 4:**
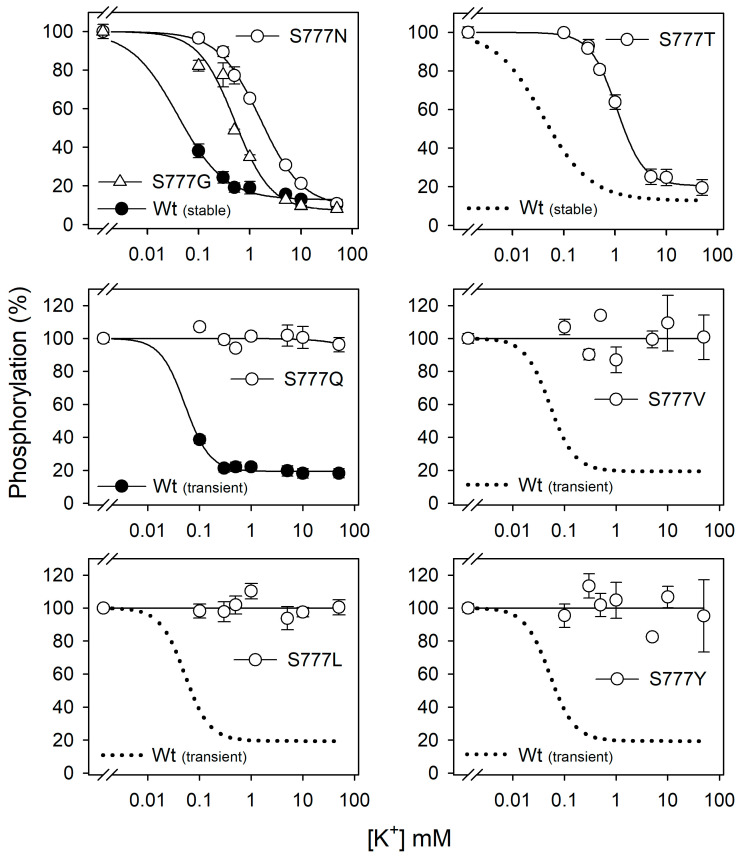
K^+^ inhibition of phosphorylation. Phosphorylation with [γ-^32^P]ATP was carried out in the presence of 50 mM Na^+^ and the indicated concentrations of K^+^. For further details, see Section 2.3. Symbols with error bars (seen only when larger than the size of the symbols) represent mean ± SEM. Each line represents the best fit of a Hill function modified to represent inhibition (Equation (3) of Section 2.5). For the mutants S777Q/V/L/Y, there was no significant inhibition at the highest K^+^ concentration tested (50 mM); thus, *K*_0.5_ is indicated as >50 mM in Table 1. Data for both the stably expressed wild type and the transiently expressed wild type are shown. Dotted lines reproduce in each panel the wild type expressed in the same way as the mutant for direct comparison.

K^+^ binding was in all the Ser777 mutants assessed by studying the ability of K^+^ to inhibit the accumulation of phosphoenzyme. This inhibition is due to the binding of K^+^ to the sites in the extracellular-facing and the cytoplasmic-facing configurations, in the respective E_2_P and E_1_ forms. In both cases, K^+^ binding drives the enzyme into the K^+^-occluded [K_2_]E_2_ form (Scheme 1, reactions 5 and 6 forward and reaction 1 backward direction of the cycle). The phosphorylation with [γ-^32^P]ATP was studied at varying K^+^ concentration in the presence of a constant Na^+^ concentration of 50 mM under conditions otherwise similar to those in Figure 3, except for the absence of oligomycin, which had been added in the experiments reported in Figure 3 to stabilize the phosphoenzyme. The results presented in Figure 4 and Table 1 show that all the Ser777 mutants exhibited defective K^+^ binding. Both the stably expressed wild type and the transiently expressed wild type showed inhibition already below 100 μM K^+^ (*K*_0.5_ approx. 50 μM). The mutants S777G/T/N were inhibited by K^+^ with apparent affinities that were reduced 11-, 22-, and 38-fold, respectively, relative to the wild type. The S777Q/V/L/Y mutants were completely insensitive to K^+^ (up to 50 mM K^+^). This indicates a decrease greater than 1000-fold in the apparent affinity for K^+^, relative to the wild type. Thus, the mutants showing the most conspicuous reduction in their apparent K^+^ affinity are the same as those that were unable to support cell growth and displayed an increased apparent Na^+^ affinity. The lack of K^+^-induced dephosphorylation of these mutants up to 50 mM K^+^ clearly indicates that their Na^+^,K^+^-ATPase activity must be very low or absent under the physiological conditions prevailing in the cell culture, thus explaining their inability to support cell growth.

### 3.5. Distribution between the ADP-Sensitive and ADP-Insensitive Phosphoenzyme Intermediates

**Figure 5:**
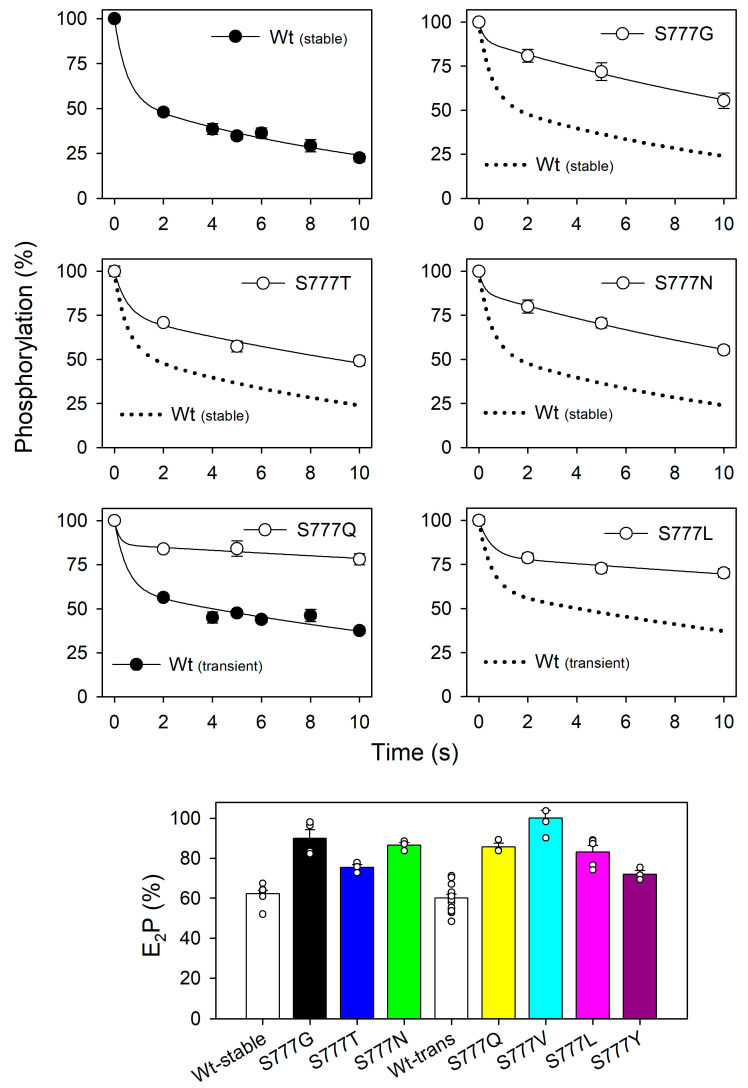
Distribution of the phosphoenzyme between E_1_P and E_2_P. Following phosphorylation with [γ-^32^P]ATP in the presence of 50 mM Na^+^, ADP was added to dephosphorylate the E_1_P fraction of the phosphoenzyme for various time intervals; see Section 2.3. Symbols with error bars (seen only when larger than the size of the symbols) represent mean ± SEM. Each line shows the best fit of a bi-exponential decay function (Equation (4) of Section 2.5) with the rapid and slow phases representing the dephosphorylation of E_1_P and E_2_P, respectively. The initial E_2_P fractions of the phosphoenzyme are shown in the column diagram and are indicated in Table 1 with statistics. Data for both the stably expressed wild type and the transiently expressed wild type are shown. Dotted lines reproduce in each panel the wild type expressed in the same way as the mutant for direct comparison.

In the wild-type Na^+^,K^+^-ATPase, two phosphoenzyme intermediates, E_1_P and E_2_P (cf. Scheme 1), can be distinguished by their differential sensitivities toward ADP and K^+^ [3,4,23]. E_1_P is highly reactive toward ADP: it rapidly dephosphorylates by transferring the phosphate back to ADP, thereby reforming ATP, but is insensitive to K^+^. E_2_P, on the other hand, is insensitive toward ADP but is highly sensitive toward K^+^, which promotes dephosphorylation via hydrolysis through binding to sites I and II in their extracellular-facing configuration. Here, we studied the ADP sensitivity of the phosphoenzyme in the absence of K^+^ but in the presence of Na^+^ to activate phosphorylation. The dephosphorylation of E_2_P is rather slow in the absence of K^+^. Upon the addition of ADP to the phosphoenzyme, two phases of dephosphorylation are observed, corresponding to ADP-sensitive (rapid phase) and ADP-insensitive (slow phase) phosphoenzyme intermediates. Figure 5 shows the dephosphorylation curves for the mutants S777G/T/N/Q/L and the wild type. The column diagram of Figure 5 and Table 1 show for all the Ser777 mutants the relative amplitude of the slow phase, reflecting the initial amount of ADP-insensitive phosphoenzyme intermediate. In all the Ser777 mutants, more of the ADP-insensitive phosphoenzyme was accumulated than in the corresponding wild type.

### 3.6. Na^+^ Dependence of Na^+^-ATPase Activity in the Active Mutants S777G, S777T, and S777N

**Figure 6:**
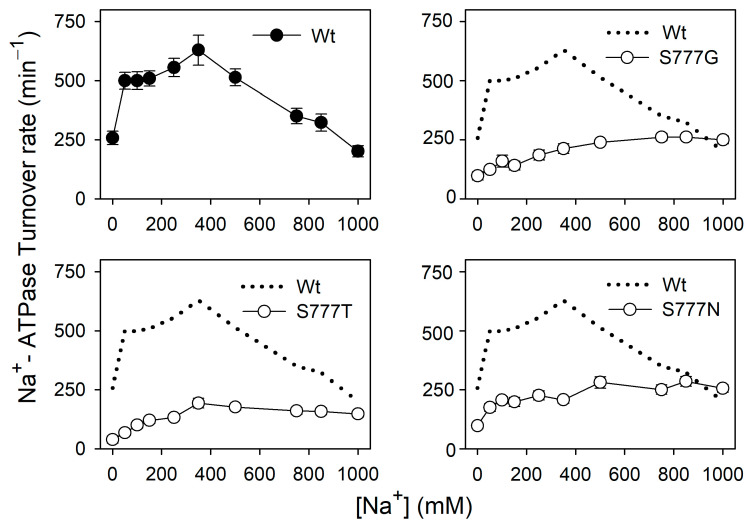
Na^+^ dependence of Na^+^-ATPase activity. ATPase activity was measured in the absence of K^+^ at the indicated concentrations of Na^+^. Symbols with error bars (seen only when larger than the size of the symbols) represent mean ± SEM. Dotted lines reproduce the wild type for direct comparison in the same panel. The catalytic turnover rate on the ordinate was calculated as the ratio between the specific ATPase activity and the active site concentration. For further details, see Section 2.2 and Section 2.3.

Figure 6 shows for the wild type and mutants S777G, S777T, and S777N that exhibited ATPase activity the so-called “Na^+^-ATPase activity” determined in the absence of K^+^. This ATPase activity is derived from the ability of Na^+^ to partially substitute K^+^ at the externally facing sites I and II of E_2_P, where it is a less potent stimulator of dephosphorylation than K^+^ [4,23,27,28,29]. Hence, Na^+^ activates the Na^+^-ATPase activity both by binding to sites I, II, and III of E_1_ from the cytoplasmic side with high affinity, stimulating phosphorylation (Scheme 1, reactions 2 and 3), and by acting as a surrogate for K^+^ by binding from the extracellular side with lower affinity to sites I and II of E_2_P, stimulating dephosphorylation (Scheme 1, reactions 5 and 6). However, high concentrations of Na^+^ usually inhibit the Na^+^-ATPase activity, as can be seen for the wild type in Figure 6. This inhibition occurs because the binding of Na^+^ from the extracellular side at site III displaces the E_1_P–E_2_P equilibrium toward E_1_P (Scheme 1, reaction 4 backward direction of the cycle). In contrast to the wild type, the mutants S777G, S777T, and S777N showed no or a strongly diminished inhibition at a high Na^+^ concentration, thus indicating that the function of site III in its extracellular-facing configuration is defective in these mutants.

### 3.7. Electrophysiological Evaluation of S777V

**Figure 7:**
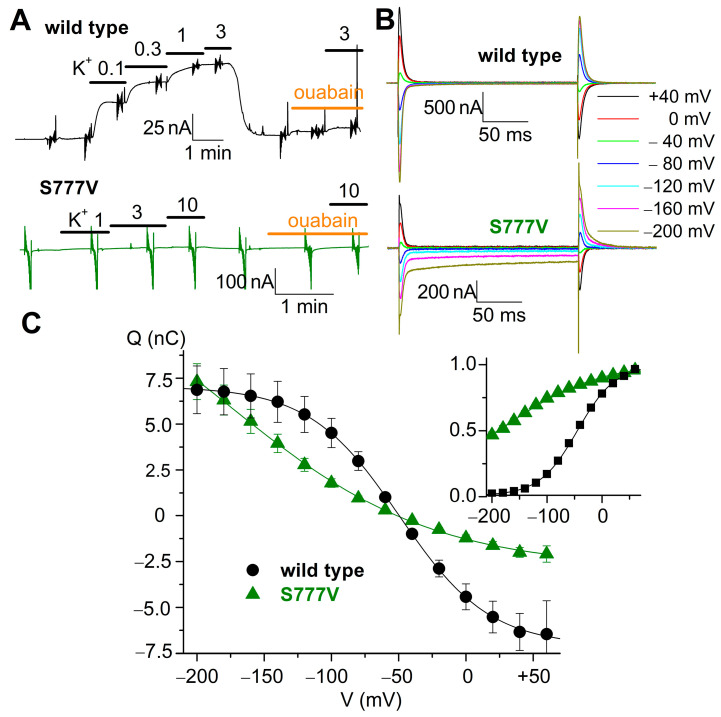
Electrophysiological evaluation of mutant S777V. (**A**): Representative steady-state currents from oocytes expressing wild type and S777V held at −50 mV and bathed in NMDG^+^ solutions. Black bars indicate application of K^+^ with the millimolar concentration indicated above the bar; orange bars indicate application of 10 mM ouabain. The vertical deflections reflect application of 100 ms long voltage pulses. No ouabain-sensitive current was observed for S777V up to 10 mM K^+^. (**B**): Representative ouabain-sensitive transient currents elicited by 200 ms long voltage pulses from −50 mV to voltages ranging between −200 mV and +40 mV in 40 mV increments, in oocytes expressing wild type and S777V. The integral of these transient currents obtained in the presence of external Na^+^, without K^+^, gives the charge moved. (**C**): Mean charge moved as a function of the applied voltage (*n* = 14 in both constructs). The solid lines represent fits of the Boltzmann distribution (shown normalized in inset), giving the parameters described in the text.

The mutant corresponding to S777N in the α2 isoform has been previously studied using electrophysiology [22], showing that this mutation markedly reduces the affinity for both Na^+^ and K^+^ on the extracellular side, as well as the turnover rate, in accordance with the findings in Figure 2, Figure 4 and Figure 6. In the present study, we applied electrophysiological measurements to Na^+^-loaded *Xenopus* oocytes under two-electrode voltage clamp to address the effect of mutation S777V on the interactions with Na^+^ and K^+^ on the extracellular side. S777V serves as an example of one of the inactive mutants with a bulky hydrophobic side chain replacing the serine side chain, resulting in a markedly increased apparent affinity for Na^+^ at the cytoplasmic-facing side (cf. Table 1). Figure 7A illustrates the currents elicited due to the application of various concentrations of K^+^ to the extracellular side in the absence of extracellular Na^+^. The wild type displayed the well-known K^+^-induced outward ouabain-sensitive current (current without ouabain—current in the presence of ouabain), reflecting the 3Na^+^:2K^+^ electrogenic exchange of the cycling Na^+^,K^+^-ATPase. In contrast, the mutant S777V failed to induce an ouabain-sensitive outward current even at 125 mM K^+^, consistent with the insensitivity of the phosphoenzyme to K^+^ seen in Figure 4.

The apparent affinity for extracellular Na^+^ was estimated by measuring the transient currents generated by voltage pulses when the Na^+^,K^+^-ATPase transits between the Na^+^-bound E_1_P and the Na^+^-free E_2_P in the absence of K^+^ (Scheme 1, reaction 4). Square voltage pulses were applied from −50 mV to voltages ranging from −200 to +40 mV, first in the absence and then in the presence of ouabain, to obtain the ouabain-sensitive transient current mediated by the Na^+^,K^+^-ATPase. Like the wild type, the mutant S777V showed a transient current, however, accompanied by a steady-state passive inward leak current (Figure 7B). Such a leak current, although more pronounced, was previously described for the α2 equivalent of S777N [22]. The parameters resulting from fitting the Boltzmann equation to the steady-state distribution of charge in individual experiments (Figure 7C) were (in mV) kT/z_q_e = 35 ± 7 and V_0.5_ = −47 ± 7 for wild type and kT/z_q_e = 84 ± 3 and V_0.5_ = −190 ± 24 for S777V (SEM of the fit, as the parameter was shared among the fits to individual experiments). V_0.5_, the center of the Boltzmann distribution, is associated with the overall apparent affinity for extracellular Na^+^, and a 20–25 mV shift to more negative potentials is equivalent to a two-fold reduction in apparent affinity [23,27,30]. On this basis, we conclude that the overall apparent affinity for extracellular Na^+^ in S777V is more than 32-fold (2^5^) reduced relative to the wild type.

## 4. Discussion

The crystal structure of the Na^+^,K^+^-ATPase in the E_1_ form with bound Na^+^ [7] has given rise to a model for the sequential and cooperative binding of Na^+^ entering the sites from the cytoplasmic side. In this model, the first Na^+^ ion transits through a tunnel formed by the empty sites I and II to reach and bind to site III. It is the occupation of site III that allows the formation of the two remaining binding cavities for Na^+^, such that site I is the second site to be occupied and site II the last. To allow Na^+^ access into site III, the conformation of Ser777 must flip, and when the Na^+^ ion at site III is bound, Ser777 coordinates this Na^+^ and at the same time contributes to making site I ready to receive the second Na^+^, i.e., Ser777 becomes a shared ligand between sites III and I. Hence, this hypothesis highlights Ser777 as a critical strategically placed residue in connection with Na^+^ binding; see also Figure 1A. Because the two conserved aspartate residues in M6 (Asp806 and Asp810 in the rat α1 enzyme) coordinate the Na^+^ in sites III and I (see Figure 1A), the occupation of these sites leads to a rotation of the unwound part of M6 that allows Asp806 to donate its free ligand side-chain oxygen to site II. The other oxygen ligands of site II are donated exclusively by residues of M4: the side chain of Glu329 and the backbone carbonyl groups of Val324, Ala325, and Val327 [7]. The rotation of M6 is an essential step in the conformational transition between the E2 and E1 forms in Na^+^,K^+^-ATPase, as well as in H^+^,K^+^-ATPase and Ca^2+^-ATPase [7,8,9,20,31]. In concert with the M6 rotation, the M5 helix is straightened, and the M4 helix displaced toward the cytoplasmic side. This allows Na^+^ to bind at site II and excludes K^+^ from binding at this site, thereby bringing the phosphorylation site aspartate in the P domain into position to receive the γ-phosphate of ATP [32].

Here, we examined the Na^+^ binding at the cytoplasmic-facing sites by analyzing the Na^+^ dependence of phosphorylation in a series of Ser777 mutants with varying side chain sizes, from glycine, the smallest, to tyrosine, one of the bulkiest residues (Figure 3). According to the van der Waals volume, the order of the residues tested in the present study is as follows: glycine (48 Å^3^) < serine (73 Å^3^) < threonine (93 Å^3^) < asparagine (96 Å^3^) < valine (105 Å^3^) < glutamine (114 Å^3^) < leucine (124 Å^3^) < tyrosine (141 Å^3^) [33]. Substitution with the three smallest residues, glycine, threonine, and asparagine, resulted in an active enzyme able to support cell growth, but with a moderate 1.4- to 7-fold reduction in the apparent Na^+^ affinity for the activation of phosphorylation, with the threonine giving the smallest effect. This is likely because it has a side chain hydroxyl group in common with serine. In the studies on the Na^+^ dependence of the Na^+^,K^+^-ATPase activity of these three mutants, the reductions in the apparent Na^+^ affinities were smaller (Figure 2), but less useful for the evaluation of specific changes in the Na^+^ interaction because the K^+^ present in the Na^+^,K^+^-ATPase assay competes with the Na^+^ at the cytoplasmic-facing sites I and II, and because the enzyme cycles through multiple conformational states.

Surprisingly, the replacement of Ser777 with large and bulky residues with a van der Waals volume > 100 Å^3^ gave rise to mutant enzymes that, although inactive and therefore unable to support cell growth, were able to be phosphorylated from ATP, and displayed a markedly enhanced apparent affinity for Na^+^, relative to the wild type, in the phosphorylation assay. The increase in Na^+^ affinity ranged from ~3-fold for glutamine to 26-fold for the bulkiest residue, tyrosine (Figure 3 and Table 1, S777Q/V/L/Y). It is reasonable to assume that the bulkiest residues prevent or at least profoundly disturb Na^+^ access to sites I and III. Therefore, a rather low apparent affinity for Na^+^, if any, would have been expected, if the occupation of these sites were obligatory for the activation of the phosphoryl transfer from ATP, as often assumed [3,4]. Hence, it appears from the present findings that phosphorylation from ATP is feasible without the occupation of sites I and III by Na^+^. Previously, we demonstrated that a Na^+^,K^+^-ATPase mutation introducing an arginine into transmembrane segment M8 (as present in H^+^,K^+^-ATPases) is compatible with Na^+^-activated phosphorylation and transport function, albeit with a stoichiometry of only 2 Na^+^ per 2 K^+^ [27]. In this mutant, the guanidine group of the arginine side chain likely functions as a tethered cation neutralizing the Na^+^ binding M8 aspartate of site III [8,27]. In a similar way, the lysine residues in M4 and M5 of a brine shrimp Na^+^,K^+^-ATPase allow cycling with a reduced pump stoichiometry of 2 Na^+^ per 1 K^+^ [34]. The present Na^+^,K^+^-ATPase mutants S777Q/V/L/Y with large and bulky neutral side chains inserted in the place of Ser777 are different because there is no tethered cation to interact with the ion-binding residues. Our demonstration that the mutants S777Q/V/L/Y can be phosphorylated by ATP is in accordance with the sequential and cooperative model based on the crystal structure [7], with site II being occupied last if it is assumed that in these mutants proper Na^+^ coordination at site II can occur without the occupation of sites I and III, and is sufficient to trigger phosphoryl transfer from ATP [31]. This would be in line with a common mechanism for the activation of phosphorylation in all P-type ATPase ion pumps, including those that possess only a single ion-binding site corresponding to site II, such as the plasma membrane Ca^2+^-ATPase [35] and the H^+^,K^+^-ATPase [36,37].

According to the sequential and cooperative model based on the crystal structure [7], the formation of site I and finally site II in the wild type depends on the occupation of site III, followed by site I, because the interaction of the first two Na^+^ ions with one of the two aspartate residues in the unwound segment of M6 (Asp810) leads to a rotation of M6 that allows the other aspartate of this segment (Asp806) to contribute to the binding of the last Na^+^ ion at site II (cf. Figure 1A). This coupling of sites was recently highlighted in mutagenesis experiments with the non-gastric H^+^,K^+^-ATPase, which could be tailored to bind Na^+^ at sites I and III, but not at site II, by replacing the lysine at the position corresponding to Ser777 in Na^+^,K^+^-ATPase with either serine or alanine. Because of the rather poor Na^+^ coordination and corresponding very low Na^+^ affinity of this construct, it was reasoned that a more optimal Na^+^ coordination at sites I and III is required to allow the full rotation of M6 needed for the proper functioning of site II in Na^+^ binding [20]. But why do we then see Na^+^ activation of phosphorylation, and even with an increase in the apparent affinity for Na^+^, in the mutants with large, bulky residues blocking sites I and III? We suggest the following explanations, which are not mutually exclusive: (1) The presence of a large and bulky residue at the position corresponding to Ser777 might cause steric effects interfering with the conformational dynamics, such that the membrane sector is locked into an E_1_-like state. This would imply that the M5 helix is straightened relative to the bent M5 helix in the E_2_ state (note that the Ser777 is located only three residues from the proline responsible for the bending of M5 in the E_2_ forms), thus resulting in a displacement of M4 toward the cytoplasmic side, which would allow the M4 Na^+^-coordinating residues to take the optimal position for Na^+^ coordination at site II. Furthermore, the unwound part of M6 would be rotated to the position where the side chain of Asp806 could participate in the coordination of Na^+^ at site II in the most optimal way [7,9,31,32]. (2) Because only one Na^+^ ion is bound (at site II), there is no electric repulsion to reduce the Na^+^ affinity, in contrast to the normal situation with repulsion between three positive Na^+^ ions bound close to each other (as recently demonstrated, the distance between the three Na^+^ ions is further diminished upon phosphorylation [9]). (3) In the absence of a Na^+^ ion at site I, the Na^+^ at site II may attract Asp806 closer, with its side chain donating two oxygen ligands to the Na^+^ binding at site II.

As an alternative to the model based on the crystal structure [7], one might speculate about a “knock-on” mechanism similar to that proposed for ion channels [38]. In such a mechanism, the three Na^+^-binding sites would be pre-formed in the E_1_ form of the wild type, and a Na^+^ ion first bound at site I might move into site III, driven by the knock-on from a Na^+^ ion binding at site II. With this binding mechanism, the three explanations given above of the increase in the apparent affinity for Na^+^ in the mutants with large, bulky residues blocking sites I and III would still be valid.

In the wild type, the E_2_P form binds K^+^ with high affinity from the extracellular side at sites I and II, whereby the dephosphorylation is promoted, leading to the K^+^-occluded [K_2_]E_2_ form (Scheme 1, reactions 5 and 6). The latter can also be reached in the backward direction of the cycle, through the binding of K^+^ at the cytoplasmic-facing E_1_ sites in competition with Na^+^ (Scheme 1, reaction 1 backward, “direct route” [3]), which likewise antagonizes the accumulation of phosphoenzyme. The mutational effects on the K^+^ dependence of phosphorylation (Figure 4) clearly demonstrate that Ser777 is a crucial residue for K^+^ binding, with even the active mutants S777G/T/N showing a considerable reduction in their apparent K^+^ affinity (up to 38-fold), and the inactive mutants S777Q/V/L/Y with the large and bulky substituents being completely insensitive to K^+^ up to 50 mM K^+^, thus explaining that their ATPase activity and transport activity is too low to support cell growth. This is corroborated by the electrophysiological experiments, showing that K^+^ being applied from the extracellular side to oocytes expressing the S777V mutant is unable to activate any outward current (Figure 7A). The latter finding is not the result of the normal 3Na^+^:2K^+^ stoichiometry having changed to electroneutral 1Na^+^:1K^+^ or 2Na^+^:2K^+^ transport, because the lack of K^+^-induced dephosphorylation in this mutant shows that the S777V mutant enzyme does not cycle.

The insensitivity to K^+^ in the inactive mutants with large and bulky substituents suggests that the presence of all the liganding groups of site II is not sufficient for K^+^ to activate dephosphorylation in these mutants, unlike the phosphorylation triggered by the Na^+^ interaction exclusively at site II. A possibility is that a proper binding of K^+^ at site I is obligatory for the activation of dephosphorylation, which would then be precluded by the mutations blocking site I. However, it has recently been reported that the activation of dephosphorylation is coupled specifically with the K^+^ occupation of site II [39]. The K^+^ binding at site II might be defective in the mutants, but not because of the absence of any of the liganding groups at site II. Rather, they are not optimally positioned for K^+^ binding due to the above-mentioned steric effect of the large and bulky S777 substituents, locking the M5 helix into the straight configuration, and the rotation of the unwound M6 segment at a position corresponding to the E_1_ form, with a preference for Na^+^ rather than K^+^. It should be noted, however, that such an effect on the conformational dynamics only pertains to the membrane part of the protein. Our studies on the distribution of the phosphoenzyme between the ADP-sensitive and ADP-insensitive intermediates show that the ADP-insensitive phosphoenzyme intermediate accumulates in all the Ser777 mutants to a larger extent compared with the wild type. Hence, the catalytic site made up of the cytoplasmic domains seems to undergo the transition from the ADP-sensitive phosphoenzyme intermediate to an ADP-insensitive intermediate, although this transition is not propagated to the membrane domain to confer K^+^ sensitivity in the inactive mutants with large and bulky substituents, as expected for a normal E_1_P-to-E_2_P transition. Such a dissociation of the events taking place in the cytoplasmic and membrane sectors has been observed previously for P-type ATPases, recently exemplified by the E_1_·Mg^2+^ structure of Na^+^,K^+^-ATPase, which appears to be intermediate between the E_1_ and E_2_ forms because the cytoplasmic sector takes an open configuration corresponding to E_1_. Meanwhile, the membrane sector is more E_2_-like, as the rotation of M6 characteristic of the E_2_–E_1_ transition is not realized with the Mg^2+^ bound in the ion-binding pocket in the membrane sector [9]. A similar hybrid state has been described for the Ca^2+^-ATPase mutant E309Q, neutralizing the critical glutamate in M4. In this mutant, the membrane sector is in the E_2_ state, whereas the cytoplasmic head piece is in an open E_1_-like state [31]. It should be added that the present criterion for the transition to E_2_P is limited to the display of ADP insensitivity, which does not necessarily require a full transition of the cytoplasmic head piece from an E_1_P type to E_2_P type of organization. Most likely, a slight increase in the distance between the bound ADP and the phosphorylated aspartate is sufficient for the ADP sensitivity to be lost.

The Na^+^ interaction at the external side was, for the active mutants S777G/T/N, examined by studying the Na^+^ dependence of the Na^+^-ATPase activity (Figure 6). The inhibition at high Na^+^ concentrations in the wild type, reflecting the binding of the Na^+^ at site III in its extracellular-facing configuration, was completely absent or strongly diminished in these mutants. This finding attests further to the importance of Ser777 in the Na^+^ binding at site III, now from the extracellular side. Furthermore, electrophysiological measurements in the oocytes of the transient currents associated with the Na^+^ binding from the extracellular side showed for mutant S777V, as an example of an inactive mutant with a large and bulky substituent, that the apparent affinity for extracellular Na^+^ was reduced more than 32-fold. This finding with S777V is in line with previous studies on the α2 variant corresponding to S777N, where similar electrophysiological measurements demonstrated a −100 mV shift in the voltage dependence of the transient currents, indicating a 16-fold reduction in the affinity for external Na^+^ [22]. Like the Na^+^ inhibition of Na^+^-ATPase activity, the transient current measurement reports on the ability of site III to bind Na^+^ from the extracellular side, thus shifting the phosphoenzyme toward the occluded [Na_3_]E_1_P intermediate. Therefore, it appears that Na^+^ site III is defective/blocked, irrespective of whether the Na^+^ enters the site from the cytoplasmic or the extracellular side of the membrane.

## 5. Conclusions

Ser777 is a crucial residue for the binding of both Na^+^ and K^+^ to Na^+^,K^+^-ATPase. Here, we have demonstrated that Ser777 mutants with large and bulky substituents, expected to prevent or profoundly disturb Na^+^ access to sites I and III, retain the ability to bind Na^+^ at site II from the cytoplasmic-facing membrane side, promoting phosphorylation with ATP, with even an increased apparent Na^+^ affinity. Hence, the Na^+^ occupation of site II is sufficient to trigger phosphorylation, with no need for Na^+^ binding at sites I and III. Furthermore, K^+^ does not activate the dephosphorylation of the ADP-insensitive phosphoenzyme formed in the mutants with large and bulky substituents, and neither Na^+^ nor K^+^ interacts properly at the extracellular-facing sites, as seen in electrophysiological studies. Taken together, these findings can be explained by the steric hindrance that excludes Na^+^ from sites I and III and interferes with the conformational dynamics in the membrane, locking the membrane sector into an E_1_-like configuration (with the unwound M6 segment rotated, the M5 helix straightened, and M4 displaced toward the cytoplasmic side). This allows Na^+^ but not K^+^ to bind at site II, while the cytoplasmic sector retains the ability to transition between ADP-sensitive and ADP-insensitive phosphoenzyme intermediates.

## Data Availability

All the presented data are in the article.
